# Gluten Deprivation: What Nutritional Changes Are Found During the First Year in Newly Diagnosed Coeliac Children?

**DOI:** 10.3390/nu12010060

**Published:** 2019-12-25

**Authors:** Maria Luisa Forchielli, Lucia Diani, Flavio Labriola, Giulia Bolasco, Alessandro Rocca, Nunzio Cosimo Salfi, Arianna Leone, Chiara Miserocchi, Laura Andreozzi, Francesca Levi della Vida, Achille Cesare Pessina, Mario Lima, Andrea Pession

**Affiliations:** 1 Paediatrics, University of Bologna, 40138 Bologna, Italy; flavio.labriola@gmail.com (F.L.); ariannaleone89@gmail.com (A.L.); francesca.levidellavida@gmail.com (F.L.d.V.);; 2 Health Science and Technologies Interdepartmental Center for Industrial Research (CIRI-SDV), University of Bologna, 40100 Bologna, Italy; 3 Dietetics and Clinical Nutrition Service, Morgagni-Pierantoni Hospital, 47121 Forlì, Italy; 4 Pathology, S. Orsola-Malpighi Hospital, 40138 Bologna, Italy; nunzio.salfi@aosp.bo.it; 5 Internal Medicine, Padua University (Retired), 35100 Padua, Italy; 6 Paediatric Surgery, University of Bologna, 40138 Bologna, Italy

**Keywords:** diet, gluten, children

## Abstract

Aim: A gluten-free diet (GFD) can expose children to excessive calories and fat intake. The study is intended to verify whether and how food intake, laboratory parameters, and growth are modified by a year of GFD. Methods: In 79 CD (coeliac disease) children (mean age 7.9 ± 3.8 years, 52 females, 27 males) diagnosed over 24 months, 24-h food diaries, food-frequency patterns, anthropometric and laboratory parameters (mainly blood sugar, insulin, lipid profile, and homocysteine) were prospectively collected before and during the first year of GFD. Nutrient intakes were compared over time and with recommendations. They were also used as regressors to explain the levels and changes of metabolic and growth variables. *p*-values < 0.05 were considered statistically significant. Results: Average macronutrient intake did not change during the year. Caloric intake remained below 90% (*p* ≤ 0.0001) and protein intake above 200% (*p* ≤ 0.0001) of recommendations. Lipid intake was stable at 34% of overall energy intake. Unsaturated fats increased (less omega-6 and more omega-3 with a ratio improvement from 13.3 ± 5.5 to 8.8 ± 3.1) and so did fibers, while folate decreased. The children who experienced a containment in their caloric intake during the year, presented a slower catch-up growth. Some differences were found across gender and age groups. In particular, adolescents consumed less calories, and females more omega-3. Fiber and simple sugar intakes emerged as implicated in lipid profile shift: fibers negatively with triglycerides (TG) (*p* = 0.033), simple sugars negatively with high-density lipoprotein (HDL) (*p* = 0.056) and positively with TG (*p* = 0.004). Waist-to-height ratio was positively associated with homocysteine (*p* = 0.018) and Homeostasis Model Assessment (*p* = 0.001), negatively with fibers (*p* = 0.004). Conclusion: In the short run, GFD is nutritionally very similar to any diet with gluten, with some improvements in unsaturated fats and fiber intake. Along with simple sugars containment, this may offer CD patients the opportunity for a fresh start. Caloric intakes may shift and should be monitored, especially in adolescents.

## 1. Introduction

A gluten-free diet (GFD) is vital for patients with coeliac disease (CD). However, it has been described as potentially unhealthy due to the extensive presence of fat and calories [1,2,3,4,5,6,7,8]. This extensive intake can change body mass index (BMI) and modify lipid profiles, with mixed results indicating potential early atherosclerosis development in adults and children, but also improvements in high-density lipoprotein (HDL) levels [9,10,11,12,13,14,15,16,17,18]. Little is known about changes in dietary habits before and during GFD. This may expose patients to dietary mistakes, further aggravated by the feeling of being deprived of gluten. For this reason, in this study, we prospectively analyzed dietary habits in children and adolescents before diagnosis and during the first year on GFD. Our focus was on the changes in dietary habits during the first year on GFD and on their consistency with recommendations. The contributory effects of dietary habits on growth and laboratory parameters were also investigated.

## 2. Methods

Patients referred to our clinic to confirm a CD diagnosis over a time span of two years were consecutively enrolled and followed for the first 12 months after diagnosis confirmation. Inclusion criteria were: age between 1 and 18 years, diagnosis of CD, and the availability of a set of measurements taken at two points in time, one before diagnosis (T0) and another 12 months after initiation of GFD (T2). The measurements include the following: (i) lipid profile (total (TC), HDL, and low-density lipoprotein (LDL) cholesterol (mg/dL) and triglycerides (TG) (mg/dL)), biochemical parameters such as blood glucose (mg/dL), thyroid and liver-function tests, iron body storage, homocysteine (mcmol/L), and Homeostasis Model Assessment (HOMA); (ii) anthropometric data (weight (grams), height (centimeters), body mass index (BMI, kilograms/meter^2^), and waist (WC) and carpal wrist circumference (cWC)). Two ratios were computed: TG to HDL, and waist to height (WHR). Besides before diagnosis (T0) and at 12 months (T2) after GFD initiation, an intermediate set of measurements was collected at 6–9 months (T1) to verify trends. Patients were considered adherent to GFD when antitransglutaminase (TTG as IgA level by ELISA: <7 U/mL), or deaminated gliadin peptide (DGP IgG by ELISA: <7 U/mL) antibodies (in case of serum immunoglobulin A deficiency), were negative. CD diagnosis was based on the Marsh/Oberhuber histological criteria [19]. 

Anthropometric data were collected under the same dress conditions and using the same measurement tools. Data collection also covered the presence of a sport activity (parent- or self-reported) and blood pressure (determined by an adequate arm size cuff sphygmomanometer). As for dietary habits, face-to-face 24-h recall diets were obtained. The strategy of a “modified” 24-h recall diet was deemed necessary to capture the patient’s habitual diet. The food-frequency interview collected the weekly consumption of meat, fish, eggs, dairy, cold cuts, pulses, fruit, vegetables, pasta, bread, cereals, and pizza. Common kitchen tools such as measuring cups, measuring spoons, food scales, or food atlases were used to detect portion sizes. Dressings, food categories (e.g., red or white meat, dairy or aged cheese, type of fish), types of drinks, and sweets were also recorded. The distinction between homemade and processed food was also recorded without distinguishing among different brands.

Food data were converted into portions and analyzed using the Food Atlas Scotti Bassani [20]. Daily average caloric intake was divided into macronutrients: carbohydrates (simple and complex), lipids (saturated vs. mono- and polyunsaturated fats, omega-6 and omega-3), and proteins. Fibers and folate were also analyzed. Comparisons were carried out with Italian recommendations (LARN) [21]. Lipid data were compared with the 50th percentile value present in the literature [22,23,24].

Means and standard deviations (SD) were used to summarize the data. The analyses were carried out using STATA Software 14 (Santa Monica, CA, USA). *t*-tests were used to compare the measurements across time and against recommendations, for the entire sample and for six subsamples based on gender and age. Finally, the metabolic and growth variables at T0 and T2, as well as their changes between T2 and T0, were regressed against the set of nutritional variables using stepwise ordinary least square option. To better approximate a normal distribution, most nutritional variables (except lipid, sugar, and omega-6 intakes) underwent a square transformation. Statistical significance was set at *p* < 0.05. Informed consent was requested for inclusion in the study. The protocol was approved by the Ethics Committee of the University Hospital (38/2017/O/Oss) and the study applied the Helsinki Declaration.

## 3. Results

A total of 79 patients (52 females and 27 males) with mean age 7.9 years ± 3.8 were identified. There were no drop-outs nor missing data. Most patients reached a negative TTG value at the end of the first year on GFD, the exceptions being 11 patients who maintained a borderline level and three who remained on the low positive side. Table 1 summarizes the data. It also shows whether T0 and T2 measurements were significantly different from each other or from recommendations. 

During the year on GFD, caloric intake decreased marginally; at the end of the first year, it was 13% below recommendations (*p* ≤ 0.001). Conversely, protein intake was 200% higher than recommendations (*p* ≤ 0.001), both before and during GFD. Lipid intake, as a percentage of overall energy intake, was 34% at both T0 and T2 and thus in line with recommendations. Overall, qualitative fat intakes overlapped recommendations: saturated fat intake went from 12.7 to 11.2% of overall energy intake while unsaturated fats increased from 18.6 to 19.2%. Dairy products and cold cuts were the main sources of saturated fat. The omega-6 to omega-3 ratio improved over the year from 13.3 ± 5.5 to 8.8 ± 3.1, falling below the 10:1 ratio commonly present in the Western diet. Carbohydrates decreased from 54% of overall daily energy intake to 52% after gluten exclusion. Simple sugars also registered a minor decrease, but still marginally exceeded the <15% recommendation. Fiber consumption improved after GFD initiation and was fairly close to recommendations (*p* = 0.08).

Overall, the variables that showed a statistically significant increase during the first year on GFD were HDL (*p* = 0.001), fibers (*p* = 0.046) and, on the anthropometric side, BMI (*p* ≤ 0.0001) and WHR (*p* = 0.01). Those that showed a statistically significant decrease over the year were unsaturated fat (*p* = 0.023), omega-6 fat (*p* = 0.0009), and folate (*p* = 0.01). TG also decreased, but without achieving full statistical significance (*p* = 0.08). 

When patients were grouped by gender, both females and males exceeded in protein intakes (*p* ≤ 0.0001). Males showed a larger reduction in caloric intake during GFD (*p* ≤ 0.0001). For both males and females, this reduction occurred only in adolescents. Over the year, both groups consumed more fibers, less omega-6 and simple sugars; females also introduced more omega-3. Niacin intakes were higher in males (*p* = 0.016). For all the other nutrition variables, the two genders were not significantly different. 

When patients were stratified by age (1–9, 10–14, and 15–18 years), statistically significant differences were fewer, as a result of the lower numbers of observations. Patients in the youngest group registered an overall reduction of folate (*p* < 0.001) and omega-6 (*p* < 0.001), as well as an increase in unsaturated fatty acids (*p* = 0.06), with a decreasing ratio of omega-6 to omega-3 fatty acids (*p* = 0.004). The decrease in folate was larger in females as compared to males (*p* = 0.04). In the intermediate age group (10–14 years), folate intake increased and the ratio of omega-6 to omega-3 fatty acids decreased; the last change being larger for females.

In Table 2, the patients’ lipid profiles are compared with the reference values. The patients are stratified by gender and age. The measurements are those taken at T2, that is, after one year of GFD. Overall, there were no significant differences from expected values. When comparing overall laboratory parameters by age stratification, HDL values increased during the GFD year (*p* = 0.001). When stratifications were done by gender, or by gender and age categories, no statistically significant differences were obtained before and after gluten exclusion. 

As some children reduced their consumption of calories during the year, a further analysis was conducted by grouping the patients according to whether their caloric intake had decreased or increased. At the end of the first year of GFD, the former had averagely increased less, both in weight (800 gr, not significant) and height (2 cm, *p* ≤ 0.001) than the latter. The same analysis was repeated, considering only the children aged 10 or less (as older children are influenced by puberty), and then again, considering only the children who had started GFD with a weight in the expected range based on the 50th percentile by age. In the first case, at the end of the first year of GFD, the children who had reduced their consumption of calories during the year averagely weighed 1.3 kg less (*p* = 0.02) and were 2.6 cm shorter (*p* = 0.01) than those who had increased their consumption of calories. No differences were detected in the second case. 

Finally, the metabolic and growth variables at T0 and T2, as well as their changes between T2 and T0, were regressed against the set of nutritional variables. HDL was negatively associated with calorie consumption (*p* = 0.04), simple sugars (*p* = 0.036), and saturated lipid amounts (*p* = 0.08). TG was associated positively with simple sugars (*p* = 0.002), and negatively with fibers (*p* = 0.033). BMI was positively associated with homocysteine (*p* = 0.000). WHR ratio was associated positively with homocysteine (*p* = 0.018) and HOMA (*p* = 0.001) levels; negatively with fiber intake (*p* = 0.004). For WC, the positive association was with HOMA (*p* = 0.001) and the negative with TC (*p* = 0.008).

## 4. Discussion

Previous studies have hypothesized that GFD leads to high caloric and fat intake and may amplify body growth and metabolism [9,10,11,12,13,14,15,16]. In particular, recent studies have stated that, to improve palatability, no-gluten pasta contains significantly higher quantities of carbohydrates and no-gluten breads have at least twice as much fat as their gluten-containing counterparts [25,26,27]. So far, however, little is known on the quantitative and qualitative nutrient changes that occur after initiation of GFD. In this study, we monitored patients before and during GFD. Strikingly, the new nutritional regimen did not lead to excessive intakes of nutrients and was even better than the gluten-containing diet. In most cases, nutrients remained within recommendations, especially for total calories, carbohydrates, fibers, and lipids. Simple sugars slightly exceeded recommendations, but they did so even before GFD. Proteins were the only nutrient to be excessively assumed but, once again, this occurred both before and during GFD: the intake was constantly more than twice the recommended amount. Animal proteins were predominant, while pulses were consumed only once a week. We could not determine the protein content of gluten-free products directly. In a survey of the nutritional profile of gluten-free products compared to standard food, gluten-free products had less protein content, with an average reduction by a factor of two in bread, pasta, and biscuits, and a factor of four in bread substitutes and rusks [28]. If this is the case, the “excessive protein intake” found in our children must have been mainly due to ingestion of animal protein food. Therefore, it must be considered a habit afflicting the general Western paediatric population rather than a GFD stigma, also considering that the overall amounts did not change before and during GFD. 

Fats and fibers are often considered the two “guilty nutrients” of a GFD. Several studies have shown a lack of fibers and a higher fat content in GFD, with an excessive amount of saturated fat [26,27,28,29,30,31]. Our children’s diets, on the contrary, showed a more appropriate intake of saturated and unsaturated fat after gluten exclusion with a favorable trend of the omega-6 to omega-3 ratio. Unfortunately, we could not be more specific in categorizing fat intake. Food labels often do not indicate qualitative amounts of fat. Studies tackling nutritional differences between GFD food and food with gluten report conflicting results. For fat contents, we have cases of GFD food with more quantities of total and saturated lipids than food with gluten; in other cases, total lipids were less represented in GFD food, but with more saturated fat when compared with gluten food [26,28]. Different country habits and regulations, inconsistent categorizations of food, and comparisons at different time points may be responsible for these dissimilarities.

Concerning dietary fibers, Barone et al. showed that CD patients consume less fibers compared to healthy adults. Similar findings were reached by other studies, both in adults and children [1,4,5,6,7]. However, opposite findings were also reported [32,33,34]. Recent food surveys have observed that fiber levels vary depending on food categories: gluten-free bread seems richer in fibers compared to gluten-containing bread, but the opposite holds for pasta [28,29,30]. In our study, fiber intake improved after GFD initiation, becoming close to Italian dietary recommendations. This pattern was evident in younger children, who are more prone to follow the instructions of parents and caregivers. Unfortunately, we were unable to categorize food and type of fibers due to the extreme heterogeneity in food brands and labels. Nevertheless, we believe that the almost appropriate amount of fibers, along with unsaturated fats, and a virtually appropriate amount of simple sugars can be considered the turning point in the healthy lifestyle application of the Mediterranean diet. Diets that are high in unsaturated fats and complex carbohydrates, including whole grains, have already been recognized as favorable in preventing several chronic preventable Western diseases. 

Besides the direct nutritional aspects of a diet change, we tried to examine the causal relationships between nutrient intakes and growth and laboratory parameters, such as lipid profile and homocysteine. Both groups of parameters can indirectly point to other nutrient deficiencies and fill the gaps left by incomplete food labels. We observed an HDL increase, which can be related to gastrointestinal improvements in digestion and absorption or to the higher intake of unsaturated fats. Similarly, the decreasing TG levels may reflect the relatively better intake of fibers along with the nearly normal intake of simple sugars. 

Hallert et al. found plasma homocysteine to be higher in adults with coeliac disease than in controls [8]. This was claimed to indicate a poor vitamin status, mainly concerning B12, B6, and folate. In our study, homocysteine values were within normal ranges, with a slightly diminishing trend similar to that of folate. Homocysteine was also associated to WHR in our children. Dicert effects cannot be further investigated in this study. Evaluation of GFD in the first year can be valuable, but also biased. This is the time when the effects of the learning curve would be more remarkable. Lack of experience, combined with fear of failure, may lead patients and parents to select repetitive and highly processed foods, which can be perpetuated for a long time. It is also the time when children may have a fast recovery, stemming simply from gluten exclusion. Follow-up data are thus required to also minimize biases during the process of recall diets or during extrapolation of nutrients from food, either when the brand is identified or a generic food category is reported, for instance, in a school menu.

Based on our results, we have introduced a “tailored” nutritional meeting with the patient and the parent or caregiver. The meeting takes place after the CD diagnosis and is intended to pass on some practical hints. For instance, to counteract excessive protein intake, the recently marketed legume-made pasta or flour, naturally without gluten, can be included in the menu. This is also a source of fiber. Feeling more at ease in selecting food, making their own food from natural ingredients, and following principles of a balanced diet will help both parents and patients become more self-confident, reach a higher quality of life, and be integrated with peers. 

The importance of studies comparing nutrient levels before and after GFD is emphasized by Melini and Melini in a recent review on GFD [35]. Further studies must be encouraged to obtain the best nutritional counselling strategy in order to improve the nutritional value of GFD and thus growth. In our study, the children who experienced an unintentional “caloric restriction” seemingly showed a slower pace of growth than those receiving more calories. Both male and female adolescents appeared to shrink their caloric intakes, perhaps due to a willingness to keep in shape or to a perceived unpalatability of GFD. However, caloric restriction during adolescence can limit growth spurts. Our group of adolescents was very small; a larger study should be carried out to verify this hypothesis.

In conclusion, the initiation of GFD does not necessarily imply a jump into bad nutritional habits. Fiber and unsaturated fat intakes seem to be improved during GFD. The key factor could be proper nutritional counselling to be addressed at time of diagnosis, when people intimately linked to pasta, pizza, and bread feel exposed and vulnerable. This dedicated time can be rewarding for all patients and their families with an eye to long-term prevention.

## Figures and Tables

**Table 1:** Summary data pre and post gluten exclusion and comparison with recommendations.

Variable		Dietary Recommendations or Normal Values	Time 0	Time 1	Time 2
		Mean ± SD or % Energy Intake	Mean ± SD	Mean ± SD	Mean ± SD
**Weight**	(kg)		27 ± 18.4	29.5 ± 19.6	32.4 ± 21
**Height**	(cm)		120.3 ± 26.7	125.4 ± 25.7	130.2 ± 25.3
**Body Mass Index (BMI)**	(kg/m^2^)		17.05 ± 4.3	17.15 ± 4.4	17.6 ± 4.7 ***
**BMI >85 percentile**	(% of total patients)		3	4	4
**Waist to Height Ratio**			0.46 ± 0.06	0.46 ± 0.06	0.47 ± 0.06
**Wrist Circumference**	(cm)		13.1 ± 1.6	13.2 ± 1.7	13 ± 1.7
**Homeostasis Model Assessment IR**			1.1 ± 0.76	1.4 ± 0.9	1.9 ± 2
**Homocysteine**	(micromole/L)	(5–15)	10.7 ± 11.3	9.2 ± 6.6	8.6 ± 3.9
**Systolic Blood Pressure**	(mmHg)		93.9 ± 11.5	97.7 ± 14.9	93.4 ± 13.2
**Diastolic Blood Pressure**	(mmHg)		57.8 ± 8.4	60 ± 6.4	57.8 ± 9.1
**Total Cholesterol**	(mg/dL)		152 ± 21.9	151.1 ± 30.2	153.4 ± 25.7
**High-Density Lipoprotein Cholesterol**	(mg/dL)		53.9 ± 14.8	56.8 ± 14.9	60.7 ± 13.4 ***
**Triglycerides**	(mg/dL)		66.4 ± 39.4	56.7 ± 21.2	53.7 ± 22.3
**Low-Density Lipoprotein Cholesterol**	(mg/dL)		88.4 ± 21.7	86.1 ± 23.7	85.5 ± 21.7 **
**Glucose**	(mg/dL)		76 ± 7.9	80.7 ± 9.4	83.7 ± 11.7 *
**Total Calories**	(Kcal/day)	1925.9 ± 504.1	1786 ± 401.8 ^^^	1703.5 ± 376.4 ^^^	1698.33 ± 377.46 ^^^
**Males <10 years**	(Kcal/day)		1502 ± 321	1455 ± 224	1510 ± 307
**Males 10–14 years**	(Kcal/day)		1833 ± 299	1913 ± 266	1880 ± 343
**Males 15–18 years**	(Kcal/day)		2197 ± 326	2143 ± 365	1960 ± 236
**Girls <10 years**	(Kcal/day)		1540 ± 250	1522 ± 215	1580 ± 231
**Girls 10–14 years**	(Kcal/day)		1955 ± 386	1995 ± 252	1966 ± 285
**Girls 15–18 years**	(Kcal/day)		1750 ± 115	2086 ± 164	1517 ± 400
**Protein**	(g/day)	26.2 ± 10.5	65 ± 21.9 ^^^	64.8 ± 19.1 ^^^	62 ± 19.3 ^^^
**Protein**	(% of energy intake)	**<15%**	14.4 ± 3 ^^	15.1 ± 3.7	14.7 ± 3.8 ^^
**Lipid**	(g/day)		66.5 ± 16.8	72.8 ± 26.25	63.65 ± 16.9
**Lipid**	(% of energy intake)	20–35 % energy	33.7 ± 5.3 ^^^	37.8 ± 8.1 ^^^	33.7 ± 5.1 ^^^
**Saturated Fat**	(g/day)		25.15 ± 8.2	25.65 ± 9.1	21.3 ± 6.5
**Saturated Fat**	(% of energy intake)	10% energy intake	12.7 ± 3.4 ^^^	13.7 ± 4.9	11.2 ± 2.6 ^
**Unsaturated Fat**	(g/day)		36.5 ± 8.65	41.3 ± 15.5	36 ± 9.9 *
**Unsaturated Fat**	(% of energy intake)	20% energy intake	18.6 ± 3.1 ^^^	21.4 ± 5.3	19.2 ± 3.2 ^^^^,^*
**Omega-6**	(mg/day)		4.3 ± 2.5	5.6 ± 4.9	2.9 ± 1.8 ***
**Omega-3**	(mg/day)		0.32±0.25	0.36 ±0.36	0.33 ± 0.25
**Omega-6: Omega-3 Ratio**		5–10:1	13.3 ± 5.5 ^	10.6 ± 9.2 ^	8.8 ± 3.1 ^^,^***
**Carbohydrate**	(g/day)		240.9 ± 62.4	202.8 ± 48.45	225.2 ± 60.2
**Carbohydrate**	(% of energy intake)	<55% energy intake	54 ± 6.8	48.4 ± 8.5 ^^^	52.9 ± 6.6 ^^^
**Simple Sugar**	(g/day)		79.3 ± 25	70 ± 21	75 ± 25
**Simple Sugar**	(% of energy intake)	<15% energy intake	17.8 ± 4.6	17 ± 4.6	17.6 ± 5.6
**Fiber**	(g/day)		12.2 ± 4	14.1 ± 5.7	13.1 ± 5.2 *
**Fiber based on Caloric Intake**	(g/1000 kcal)	8.4 g/1000 kcal	7.4 ± 1.6 ^^^	8.3 ± 2.6	8.2 ± 3.6
**Folate**	(microgr/day)		155.5 ± 66.6	121.4 ± 57.1	118.4 ± 63.5 ***

Time 0: time before coeliac disease (CD) diagnosis; Time 1: 6 months after gluten-free diet (GFD) initiation; Time 2: one year after GFD initiation. SD: standard deviation. Recommended calorie and protein intakes were computed based on child age and gender and then averaged. For calories, the Schofield equation plus a median activity factor of 1.39 < 3 years, 1.57 between 3 and 10 years, and 1.73 > 10 years was estimated based on Scientific Advisory Committee on Nutrition. Dietary recommendations for energy. 2011. For protein intake, the average requirement was used. Statistically significant differences of T2 measurements: from time 0 at *p* < 0.05 (*), *p* < 0.01 (**), *p* < 0.001 (***); from recommended intake at *p* < 0.05(^), *p* < 0.01 (^^), *p* < 0.001 (^^^).

**Table 2:** Comparison between expected [22,23,24] and detected mean lipid profile values after the first year of gluten exclusion.

	Total Cholesterol					High-Density Lipoprotein Cholesterol					Triglycerides					Low-Density Lipoprotein Cholesterol				
Age and Gender Group	AAP	IDEFICS	Detected	Δ	Δ	AAP	IDEFICS	Detected	Δ	Δ	AAP	IDEFICS	Detected	Δ	Δ	AAP	IDEFICS	Detected	Δ	Δ
**(*n*. pts)**	mg/dL	mg/dL	mg/dL	%	95% CI	mg/dL	mg/dL	mg/dL	%	95% CI	mg/dL	mg/dL	mg/dL	%	95% CI	mg/dL	mg/dL	mg/dL	%	95% CI
**Male**																				
1–9 (16)	153	151.6–155.5	148 ± 28	−3.3	−21	55	44.8–56.2	60 ± 12	9.0	−3	48	45	47 ± 17	−2.1	−12	90	93–86	76 ± 23	−15.5	−31
					+10					+11					+8					+1
10–14 (7)	161	156.1–156.4 *	153 ± 29	−5.0	−28	55	55.6–55.8 *	67 ± 12	21.8	−4	58	45 *	45 ± 23	−22.4	−42	94	84.8–84.2 *	73 ± 13	−22.6	−43
					+46					+28					+15					−1
15–18 (4)	152	NA	131 ± 8	−13.8	−36	46	NA	52 ± 12	13	−15	68	NA	46 ± 9	−32.3	−36	93	NA	70 ± 9	−24.7	−38
					−6					+25					−8					−9
**Female**																				
1–9 (38)	164	153.9–161.0	159 ± 29	−3.0	−16	52	43.8–54.8	53 ± 13	1.9	−4	57	48	72 ± 37	26.3	+0.5	98	96.8–92.3	93 ± 23	−5.1	−15
					+5					+6					+27					+2
10–14 (12)	159	162.2–162.8 *	157 ± 13	−1.6	−9	52	54.5–54.3 *	60 ± 11	15.4	−1	68	48 *	63 ± 21	−7.3	−18	94	91.5–91.1 *	85 ± 12	−9.6	−16
					+9					+15					+10					+2
15–18 (2)	157	NA	160 ± 21	2.5	−137	51	NA	71 ± 2	39.2	+0.4	64	NA	43 ± 24	−32.8	−109	93	NA	81 ± 18	−13	−93
					+193					+38					+195					+153

*: reference valid until 11 years of age; NA: not available; AAP: American Academy of Paediatrics [23,24]; IDEFICS: European consortium. Blood lipids at the 50th percentile [24]; Δ: percentage difference between the detected and AAP values.
